# Wireless Torque and Power Transfer Using Multiple Coils with LCC-S Topology for Implantable Medical Drug Pump

**DOI:** 10.3390/s21238150

**Published:** 2021-12-06

**Authors:** Jaewon Rhee, Yujun Shin, Seongho Woo, Changmin Lee, Dongwook Kim, Jangyong Ahn, Haerim Kim, Seungyoung Ahn

**Affiliations:** 1 The Cho Chun Shik Graduate School of Green Transportation, Korea Advanced Institute of Science and Technology, Daejeon 34051, Korea; elly0386@kaist.ac.kr (J.R.); yujun.shin@kaist.ac.kr (Y.S.); seongho@kaist.ac.kr (S.W.); ckdals4707@kaist.ac.kr (C.L.); jangyong.ahn@kaist.ac.kr (J.A.); haerim@kaist.ac.kr (H.K.); 2 Department of Automotive Engineering, Yeungnam University, Gyeongsan 38541, Korea; dwkim@yu.ac.kr

**Keywords:** wireless power transfer (WPT), wireless torque transfer (WTT), inductive coupling, battery-less, implantable medical device (IMD), drug pump

## Abstract

In this paper, we propose a method of wirelessly torque transfer (WTT) and power (WPT) to a drug pump, one of implantable medical devices. By using the magnetic field generated by the WPT system to transfer torque and power to the receiving coil at the same time, applications that previously used power from the battery can be operated without a battery. The proposed method uses a receiving coil with magnetic material as a motor, and can generate torque in a desired direction using the magnetic field from the transmitting coil. The WPT system was analyzed using a topology that generates a constant current for stable torque generation. In addition, a method for detecting the position of the receiving coil without using additional power was proposed. Through simulations and experiments, it was confirmed that WTT and WPT were possible at the same time, and in particular, it was confirmed that WTT was stably possible.

## 1. Introduction

Recently, with the rapid development of wireless power transfer (WPT) technology, it has been commercialized in various devices and is applied to various fields. WPT system can increase the degree of freedom of devices that have been supplied with power through the wire, and can solve both the inconvenience and the safety problem of the wire at the same time. WPT technologies ranging from low-power such as mobile phones and medical devices to large-power such as electric vehicles and industrial robots are being developed and in progress [1,2].

The magnetic resonance type WPT system that is mainly used is a technology that transmits power with relatively high efficiency using magnetic coupling between transmitting coil (TX coil) and receiving coil (RX coil) by reducing impedance at resonance frequency. A lot of research is underway to increase the efficiency and the gap between the TX coil and RX coil so that it can be applied to various applications [3,4].

This WPT technology has made a breakthrough, especially in the field of medical devices related to human life. With the accelerated aging of the population and the development of medical technology, the development of electrically powered active implantable medical devices (IMD) is increasing. Therefore, research on wirelessly supplying power from outside the body to the inside using WPT technology is being actively conducted [5,6]. Most of the conventional papers are studies on wireless charging of batteries using secondary batteries to reduce the burden of replacing batteries [7,8].

However, wireless charging of batteries has the ultimate problem of requiring medical revision surgery for replacement or removal when the battery is nearing the end of its life. Therefore, there is a need for a system that can be used immediately by receiving power wirelessly from the outside without a battery, which is expected to relieve the physical and economic burden of many elderly patients.

There are several active IMDs commercially available, such as pacemakers, electrocardiogram (ECG) sensors, drug pumps, and cochlear implants. Among them, the device to be dealt with in this paper is the drug pump. This medical device is designed to inject drugs into the body at a fixed time through a catheter connected to a pump because it is difficult to inject narcotic pain relievers into the spinal cord every time for patients with severe chronic pain [9]. The device has a separate drug storage bag, and when the drug is out, patient can visit the hospital once every 2–3 months to easily refill the drug by subcutaneous injection.

For the operation of the conventional drug pump, the motor is driven using the power from the battery, however, research on generating torque only with an external power source without a battery is required. The torque required to drive the drug pump is about 4.8 μNm for commercial products [10]. Therefore, the method proposed in this paper can receive power while generating the required torque using the magnetic field generated when using the WPT system. However, it can also be used to charge a backup battery with a smaller capacity than conventional using the received power. This can increase user convenience and can be applied to more diverse patients.

In previous works, there are research that drive a motor or transmit force using WPT technology. First, there is a research using WPT technology to wirelessly supply power to a heart pump, an implantable medical device, to drive a DC motor [11]. In [11], the RX coil to be inserted into the body and a separate motor are required. Therefore, the proposed research proposes a method using the RX coil as a motor, unlike previous research. However, there are also research on applications requiring low power, such as medical devices [12]. In [12], There is research to move the micro robot in a specific direction using WPT technology in the body. However, this research generates a force in a specific direction to drive the micro robot. Since the robot moves inside the body based on the TX coil outside the body, there is a limit to the rotational motion that generates torque. Specifically, this research uses one TX coil, which can only generate torque and rotate when placed at an angle of less than 90 degrees. After rotation, the RX coil moves in the direction of the TX coil by the magnetic field. However, in order to drive the drug pump, a structure that can rotate 360 degrees is required, and it should not move in the direction of the TX coil even after rotation. In addition, there is a limitation in that the volume of the system is too large by arranging the TX coil on the side of the RX coil to generate rotational force in the past.

Therefore, in this paper, by combining the two main topics of wireless torque transfer (WTT) and WPT, we propose a method for operating a drug pump with an external power source without a battery and the planar type structure to solve the conventional volume problem. If WPT is possible, the received power can be used in various ways, such as operating a flow meter for flow rate sensing or using it for communication with the outside of body. In addition, the actual battery of commercialized drug pump occupies about one-third of the drug pump’s volume. Thus, by removing the battery, the volume of the device can be reduced or the drug storage capacity can be increased.

The research to be introduced in this paper as follows. The magnetic field generated in the WPT system is used to perform WTT. Specifically, the direction of the magnetic field formed according to the on/off of the multiple TX coils were analyzed. In addition, a method for generating a desired torque by analyzing a parameter electrically related to the generated torque is proposed. WPT system was also applied using the LCC-Series topology to generate more stable torque. Finally, a method of sensing the position of the receiver in the body was introduced to control the system using only electrical parameters outside the body.

The composition of the paper is as follows. First, in the analysis, the coil structure of the proposed system is explained, and how WPT and WTT are possible at the same time, respectively. In addition, we propose a method of detecting the receiving coil for system control. Next, in simulation and experimentation, the proposed method was verified, and finally there is discussion and conclusion about the results.

## 2. Analysis of WTT and WPT for Implantable Medical Device

In the analysis part, we propose a method for operating an implantable medical de-vice without a battery by WTT and WPT at the same time using electromagnetic induction between TX coils and RX coil. First, the structure of the proposed IMD, the drug pump, will be described, and the method of WTT and WPT will be described. Additionally, there is a description of a method for detecting the position of the pump for driving the pump.

### 2.1. Proposed WTT and WPT System

**Figure 1:**
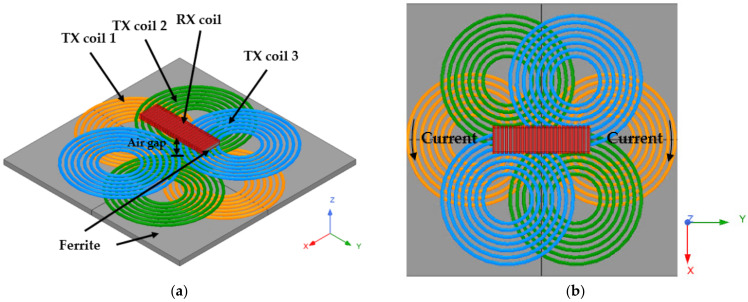
Design of the proposed models for WTT and WPT. (**a**) Perspective view; (**b**) Top view. Three TX coils are stacked at intervals of 60 degrees. RX coil is wound in the form of a solenoid.

The proposed model can be divided into a receiver implanted in the body and a transmitter located outside the body. Among the receivers implanted in the body, the RX coil should act as a pump to deliver drugs while receiving power. As shown in Figure 1, the receiver is designed in the form of a coil wound around ferrite. The ferrite of RX coil is aligned by an external magnetic field to generate torque and increase the magnetic field induction to increase the power transfer efficiency of WPT. Although various models of drug pumps are on the market, Medtronic’s SynchroMed 2 model is generally inserted into the human body in the form of a cylinder with a diameter of about 80 mm and a thickness of about 20 mm [13]. The size of the RX coil was designed to fit the size of a commercially available drug pump, and it was designed with a ferrite sheet to reduce the weight of the magnetic material to facilitate torque generation. Therefore, the designed RX coil can be sufficiently implanted into the human body, and the implanted RX coil has the advantage of receiving power while replacing the motor. 

The transmitter of the designed model is composed of three TX coils and ferrite to transmit power and torque at the same time, and each coil is composed of a pair of DD coils [14]. Although the DD coil is a single coil, it reverses the winding direction like two coils joined together. It induces a magnetic field horizontally, and has strong to misalignment. Therefore, the DD coil is suitable for generating torque in the xy plane based on the z-axis because the current in one coil flows oppositely as shown in Figure 1, so the vertical magnetic field is canceled and the horizontal magnetic field is increased. The detailed explanation is continued in Section 2.2.

In addition, as shown in Figure 1, each of the coils of the same color represents one RX coil, and is spaced apart by 60 degrees from each other, and the coils are not connected to each other. Each coil is called TX coil 1, TX coil 2, and TX coil 3 in the order close to the ferrite. Like the receiver, the ferrite under TX coils is arranged in the form of a pad to increase the guiding of the magnetic field from the TX coils.

### 2.2. Methodology of Wireless Torque Transfer

The fundamental principle of the proposed WTT method is the alignment of ferrites through the guiding of magnetic field. In order to transmit torque to the RX coil of the designed model, the surrounding magnetic field must be generated in the desired direction. At this time, the magnetic field is generated by the current flowing in the coil, and in particular, the magnetic field generated by the current flowing in TX coils which are larger than RX coil, is dominant. Specifically, it generates torque by using the property of magnetic materials to be aligned to minimize magnetic potential energy under a magnetic field [15]. Therefore, when power is applied to a desired TX coil according to the position of the RX coil, the ferrite of the receiver rotates in the center direction of the turned-on TX coil.

**Figure 2:**
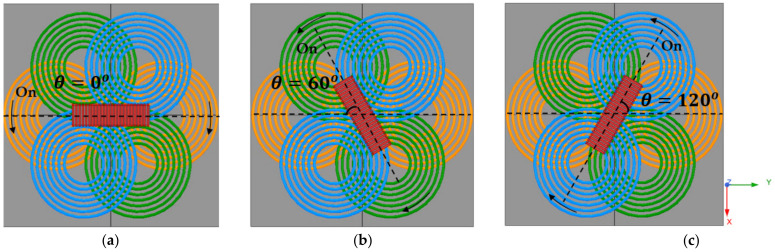
This figure shows the rotation of the receiving coil according to the on/off of the TX coil. The arrow on the coil is the direction of the current. When the TX coil is turned on sequentially from 1 to 3, the receiving coil rotates clockwise. (**a**) Alignment direction of RX coil when TX coil 1 is on; (**b**) Alignment direction of RX coil when TX coil 2 is on; (**c**) Alignment direction of RX coil when TX coil 3 is on.

As shown in Figure 2, the RX coil is aligned in the same direction as the turned-on TX coil 2. In the initial state of Figure 2a, when TX coil 2, which is the green color, is turned on, the RX coil rotates clockwise by 60 degrees to align in the same direction as this coil. Similarly, as shown in Figure 2c, when TX coil 3, the blue one, is turned on, the RX coil rotates in the direction aligned with TX coil 3. Therefore, it is possible to wirelessly transmit torque to the RX coil by controlling the magnetic field in the desired direction.

In the end, if the TX coil is turned on sequentially in one direction in the proposed model, a rotating magnetic field is generated based on the z-axis, and the RX coil can also rotate according to the direction of the magnetic field. At this time, AC current is applied to each TX coil in order to use WPT technology later, the direction of the magnetic field changes with time on the xy-plane. Therefore, if the axis is fixed so that it does not move in the z-axis direction, the receiving coil rotates clockwise or counterclockwise on the xy-plane and torque is generated. 

**Figure 3:**
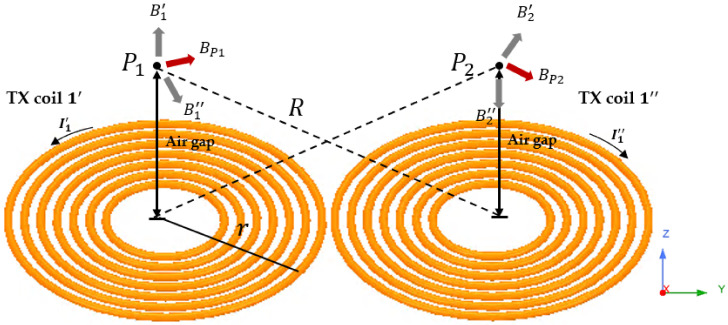
Magnetic field generation direction when TX coil 1 is on. The figure shows the analysis of the magnetic field when only TX coil 1 is turned on among the three TX coils. One DD coil is divided into two and analyzed.

Figure 3 shows the configuration when only TX coil 1, which is at the bottom of the three TX coils, is turned on to confirm the magnetic field change when the transmitting coil is turned-on. The TX coil is in the form of a DD coil, but for the purpose of analysis, it is assumed that two coils of the same shape and number of turns and only current flowing in reverse are arranged on the same plane. Since a current of the same magnitude flows through the two coils with a phase difference of 180 degrees, the current flowing through each coil can be expressed as the Equations (1) and (2).

(1) $$I_{1}^{\prime }=I_{1M}^{\prime }\sin\omega t\;$$

(2) $$I_{1}^{\prime \prime }=I_{1M}^{\prime }\sin\left(\omega t+\pi \right)=-I_{1}^{\prime }$$

$i_{1M}^{\prime },\;\omega$ and t are the maximum current value of TX coil 1′, angular frequency and time, respectively.

The magnetic field at any point P_1_ away from the central axis of the coil with the largest magnetic field strength can be obtained as the sum of the magnetic fields due to the current flowing through the two coils. First, the magnetic field generated in P_1_ by TX coil 1′ is generated in the z-axis direction and can be obtained as Equation (3) [16].

(3) $$\vec{B_{1}^{\prime }}=\frac{\mu_{0}NI_{1}^{\prime }}{2r}\;\vec{a_{z}}=B_{z1}^{\prime }\vec{a_{z}}$$

$\mu_{0},N$ and r are the permeability of free space, the number of turns of each coil, and the radius of the coil. Since the x and y direction magnetic field component is canceled during integration according to Biot-Savart law, only the z-direction component appears as shown in Equation (3).

The magnetic field generated in P_1_ by the TX coil 1″ is expressed as the sum of the +y-direction component and the -z-direction component because the current direction of the TX coil 1″ is opposite to the current direction of the TX coil 1′. This can be expressed as Equation (4).

(4) $$\vec{B_{1}^{\prime \prime }}=B_{y1}^{\prime \prime }\vec{a_{y}}-B_{z1}^{\prime \prime }\vec{a_{z}}$$

In Equation (4), $B_{z1}^{\prime \prime }$ and $B_{y1}^{\prime \prime }$ are the magnitudes of each component that are both positive magnitudes of each component, and the direction of magnetic field is shown in Figure 3. Therefore, the total direction of the magnetic field generated by the TX coil 1 is determined by the sum of the y-direction and z-direction components.

(5) $$\vec{B_{P1}}=\vec{B_{1}^{\prime }}+\vec{B_{1}^{\prime \prime }}=B_{y1}^{\prime \prime }\vec{a_{y}}+\left(B_{z1}^{\prime }-B_{z1}^{\prime \prime }\right)\vec{a_{z}}$$

The magnetic field component at an arbitrary point P_2_ away from the center of the TX coil 1″ by an air gap can be obtained in the same way as above, and the total magnetic field is also expressed as the sum of the y and z component. At this time, in the drug pump, it is assumed that the RX coil acting as the motor of the pump is fixed in the z-axis direction, so the magnetic field that actually affects the torque can be regarded as only the component in the y-axis direction.

Applying the above analysis to the situation where the coils are turned on one by one like the mechanism suggested above, and assuming that the magnetic field in the z-axis direction does not affect the torque generation, the magnetic field is rotated as follows. First, the Equation (6) is the magnetic field when only TX coil 1 is turned on.

(6) $$\vec{B_{1}}=B_{1}\vec{a_{y}}$$

When the proposed model is viewed from the top, when the next TX coil is turned on sequentially, the magnetic field direction rotates clockwise, which can be expressed using the rotation matrix.

(7) $$R=\left[\begin{array}{cc} \cos\theta & -\sin\theta \\ \sin\theta & \cos\theta \end{array}\right]$$

where

(8) $$\theta =\frac{\pi }{3}\;\left[rad\right]$$

Since each TX coil is arranged at intervals of 60 degrees, using the rotation matrix to find the magnetic field when turning on the remaining TX coils except for TX coil 1 is as shown in Equations (9) and (10). As in Equation (6), the magnetic field considers only the components of the xy-plane that affect the torque [17].

(9) $$\vec{B_{2}}=B_{2}\left(\frac{\sqrt{3}}{2}\vec{a_{x}}+\frac{1}{2}\vec{a_{y}}\right)\;$$

(10) $$\vec{B_{3}}=B_{3}\left(\frac{\sqrt{3}}{2}\vec{a_{x}}-\frac{1}{2}\vec{a_{y}}\right)$$

From Equations (6), (9), and (10), the magnetic field generated by the TX coil in the xy-plane where the RX coil is located rotates clockwise. 

When the RX coil surrounding the ferrite is positioned in such a magnetic field, the receiver is aligned according to the direction of the magnetic field. Since ferrite is a soft magnetic material, it has a property of being aligned according to an external magnetic field. If the receiver is made of permanent magnets instead of ferrites, the polarity is not changed by an external magnetic field, so there is no torque and it only vibrates. This is why ferrite, which is a soft magnetic material, was chosen for torque generation. 

If so, how the torque will generate under the above magnetic field is as follows. The torque is generally proportional to the magnetic field and the magnetic moment, and can be obtained as the cross product of the two vectors as shown in Equation (11) [16].

(11) $$\vec{T}=\vec{m}\times \vec{B}$$

In Equation (11), the magnetization vector $\vec{m}$ can be expressed as the product of the magnetic field strength and the magnetic susceptibility, so Equation (11) is summarized as Equation (12) [15]. In Equation (12), α is the angle between the magnetization vector and the magnetic field vector.

(12) $$T=\frac{1}{2}\left|m\right|\left|B\right|\sin\left(2\alpha \right)\sin^{2}\left(\omega t\right)=\frac{1}{2}\chi \mu {\left|H\right|}^{2}v\sin\left(2\alpha \right)\sin^{2}\left(\omega t\right)≅\;\;\;\;\;\;\;\frac{1}{2}\mu_{0}\mu_{r}^{2}{\left|H\right|}^{2}\sin\left(2\alpha \right)\sin^{2}\left(\omega t\right)$$

where

(13) 0≤ωt≤π

(14) 0≤α≤π/2

(15) $$\mu_{r}\gg 1$$

χ,v,μ,$\;\mu_{0}$, and $\mu_{r}$ are the susceptibility, unit volume, permeability, permeability of vacuum, and the relative permeability, respectively. Using the relationship between permeability and magnetic susceptibility and the condition that the specific permeability of ferrite is sufficiently greater than 1, the torque is the same as Equation (12). So, the torque is proportional to the square of the permeability and magnetic field strength. In addition, since the polarity of ferrite continues to change according to the external magnetic field, the torque is generated only in one direction during one cycle. Therefore, a condition such as Equation (13) is added.

(16) H ∝NI 

(17) $$T\;\propto \mu_{r}^{2}N^{2}I^{2}$$

Since the magnetic field strength is proportional to the number of turns and the current according to the Ampere law, the torque is ultimately proportional to the square of the relative magnetic permeability, the number of turns, and the current. Therefore, in order to generate a large torque, a magnetic material having a large relative magnetic permeability is used, and the number of turns and current of the coil are increased.

### 2.3. Method of Wireless Power Transfer

The proposed method uses magnetic resonance WPT technology to wirelessly transmit torque and power at the same time. It is a technology that uses resonance to transmit power through magnetic coupling of the transmitter and receiver. By the time-varying magnetic field generated from the TX coil, an electromotive force is induced in the receiving coil according to Faraday’s law and used as a voltage source, and thus power is transmitted to the load. 

As described in Section 2.2, torque is proportional to the square of the relative magnetic permeability, the number of turns, and the current. Therefore, the variable that can adjust the torque is the current. Since dynamic WPT is performed in the proposed structure, the current flowing in the TX coil greatly changes due to the change of mutual inductance in the general series-series topology. In addition, in the series-parallel topology, since the compensation capacitance of the TX part changes due to the change of mutual inductance, a constant current compensation circuit that is not affected by the receiving side is required except for the two representative topologies (series-series, series-parallel) [18].

**Figure 4:**
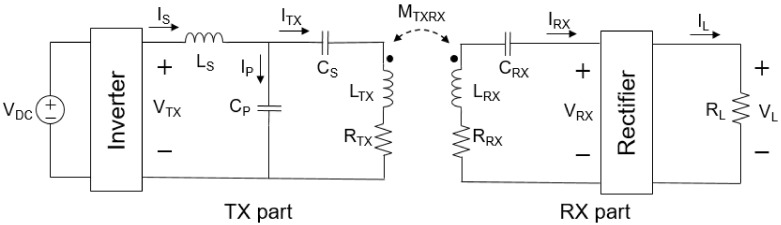
Simplified circuit of the proposed system. To generate constant current, the TX part uses an LCC topology, and the RX part uses a series topology to lower the complexity of the system. The circuit is shown from the DC input to the load.

LCC-series topology is used as shown in Figure 4 by using an LCC circuit at the TX part and a series circuit at the RX part so that the current flowing through the TX coil becomes a constant current regardless of the change in mutual inductance. The electrical parameters of the circuit of the proposed system are shown in Table 1. The DC voltage source passes through the inverter and becomes an AC voltage and a constant current flow through the TX coil under the resonance condition of the equation below. Specifically, when a DC source is input, a square wave voltage is output from the inverter by the operating frequency. As it passes through the capacitor of the LCC topology, then the DC component is blocked and a constant sinusoidal AC current flows in the TX coil regardless of RX part. In Equation (18), $C_{P}$, which is the parallel capacitance of the TX part, and the operating frequency do not change, and $L_{S}$ is also fixed. Therefore, $I_{TX}$ changes only by the input voltage [19].

(18) $$I_{TX}=-j\omega_{0}C_{P}V_{TX}$$

where

(19) $$\omega_{0}=\frac{1}{\sqrt{L_{S}C_{P}}}$$

In addition, the proposed LCC-Series topology is a situation when only one of the three TX coils is turned on. $L_{S}$ and $C_{P}$ are always fixed, but the size of $C_{S}$ is different for each TX coil due to resonance conditions. In $C_{S}$, a resonance condition is presented by setting the apparent power on the input side to 0 in order to minimize the reactive power. Although the three TX coils are designed with the same number of turns and dimensions, the inductance is different due to the difference in distance from the ferrite, and therefore the $C_{S}$ of each TX coil is different. Equation (20) is an expression to obtain $C_{S}$ under the resonance condition of Equation (21) [19].

(20) $$C_{S}=\frac{1}{\omega_{0}^{2}(L_{TX}-L_{S})}\;$$

where

(21) $$\omega_{0}=\frac{1}{\sqrt{C_{S}(L_{TX}-L_{S})}}$$

The fundamental principle of the proposed WTT method is the alignment of ferrites through magnetic field guiding. In order to transmit torque to the receiver of the designed model, the surrounding magnetic field must be generated in the desired direction. The magnetic field is generated by the current flowing in the coil, and in particular, the magnetic field generated by the current flowing in the relatively large TX coil than RX coil is dominant. Therefore, when power is applied to a desired TX coil according to the position of the RX coil, the ferrite of the receiver rotates to the direction of turned-on TX coil [20].

(22) $$\eta =\frac{P_{out}}{P_{in}}=\frac{{\left(\omega_{0}M\right)}^{2}}{R_{TX}R_{L}+{\left(\omega_{0}M\right)}^{2}}\;$$

where

(23) $$R_{L}\gg R_{RX}$$

In the LCC-Series topology, the efficiency is the same as Equation (22), which is when $R_{RX}$ is small enough for the load resistance. Therefore, in the above system, the efficiency is determined by the mutual inductance between the transmitting and receiving coils and the equivalent series resistance of the TX coil. In general, the coil resistance is much smaller than the load resistance, and the load resistance is much larger than the coil resistance in the proposed system.

Therefore, using the LCC-Series topology, torque can be transmitted relatively uniformly regardless of the mutual inductance of the TX coil and RX coil, and the power transfer efficiency varies according to the mutual inductance. Thus, the power delivered to the load depends on the position of the RX coil. 

### 2.4. Method of Receiving Coil Detection

The proposed method consists in the order of turning on the next TX coil according to the position of the RX coil to operate the pump in the desired direction. Therefore, in order to prevent the drug from flowing back, it is necessary to determine which of the three TX coils to turn on when operating the pump.

Mutual inductance or coupling coefficient, which indicates the degree of coupling between the TX coil and RX coil, is a parameter to determine the position of the RX coil. However, since the RX coil is located in the body, it is difficult to directly measure the parameter, and thus there is a problem in that additional elements such as a sensor or a communication device are required, thereby increasing the complexity of the system. Therefore, it is necessary to determine the position of the RX coil with information from the TX part, which has a relatively flexible measurement environment, without using additional sensors or communication devices. 

**Figure 5:**
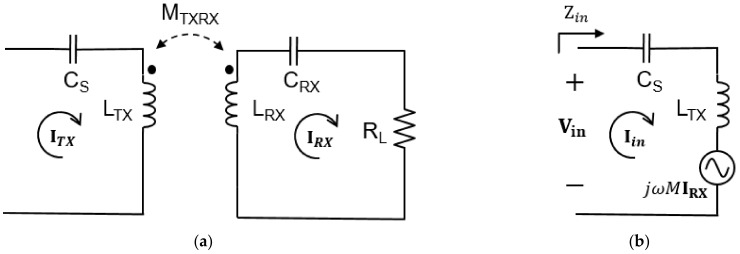
RX coil detection method of the proposed WTT and WPT system using reflected resistance. (**a**) Simplified circuit from the TX coil to RX part; (**b**) Equivalent TX part circuit reflecting RX part. It is possible to check the impedance change of the TX coil by the change of the voltage reflected on the TX part by mutual inductance.

For this, the equivalent reflection resistance of the TX coil may be used. In Figure 5, Mutual inductance changes according to the location of the RX coil, and accordingly, the magnitude of the voltage source reflected in the TX side changes. If the equivalent resistance change of the TX coil can be measured using this principle, the position of the TX coil can be found relatively conveniently.

The impedance reflected on the TX part can be obtained as in Equation (24) through the voltage reflected on the TX part and the current flowing through the coil. At this time, the current flowing through the RX coil is the same as Equation (25), and the inductance and compensation capacitor of the RX coil disappear due to the resonance condition.

(24) $$Z_{in}=\frac{V_{in}}{I_{in}}=\frac{j\omega_{0}MI_{RX}}{I_{TX}}=\frac{{\left(\omega_{0}M\right)}^{2}}{R_{L}}\;$$

where

(25) $$I_{RX}=\frac{j\omega_{0}MI_{TX}}{j\omega_{0}L_{RX}+\frac{1}{j\omega_{0}C_{RX}}+R_{L}}$$

(26) $$\omega_{0}=\frac{1}{\sqrt{L_{RX}C_{RX}}}$$

If the resonance condition of Equation (26) is applied, ideally only the real part of the input impedance remains. Therefore, the equivalent resistance reflected by the TX coil can be expressed as Equation (27) [21].

(27) $$Re\left|Z_{in}\right|=\frac{{\left(\omega_{0}M\right)}^{2}}{R_{L}}+R_{TX}\;$$

$Re\left|Z_{in}\right|$ is the sum of the resistance of TX coil and real part of the impedance reflected by the RX part. Therefore, since the equivalent series resistance of the TX coil changes in proportion to the square of the mutual inductance, the approximate position of the RX coil can be known by measuring the resistance of the TX coil. At this time, the smaller the size of the load resistance, the larger the equivalent series resistance of the TX coil changes according to the change of the mutual inductance. Therefore, the larger the mutual inductance between the TX coil and RX coil, that is, the more aligned with the turned-on TX coil, the larger the equivalent resistance of the TX coil is measured. 

## 3. Simulation

Verification through simulation is largely divided into WPT and WTT. The simulation step is as follows. First, the proposed structure is modeled using ANSYS Maxwell Electronics, a finite element method solver, and the inductance of the coil is obtained.

After that, the LCC-S topology circuit is modeled, and the compensation capacitance and additional inductance are obtained through the previously obtained coil inductance, and then the circuit simulation is performed. In addition, the load resistance of the system is 20 ohms. In this process, the system power transfer efficiency by position of the RX coil can also be additionally checked.

Lastly, after inputting the TX coil and RX coil currents extracted from the circuit simulation result back to Ansys Maxwell Electronics, the torque for each angle is calculated using a transient solver. The operating frequency of the system is 125 kHz according to ETSI EN 302 195-2 (Radio equipment in the frequency range 9 kHz to 315 kHz for Ultra Low Power Active Medical Implants (ULP-AMI) and accessories) [22].

### 3.1. Simulation Setup

**Figure 6:**
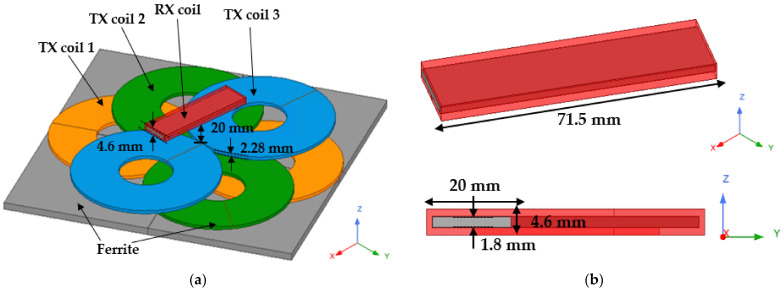
Simulation setup of the proposed models for WTT and WPT. (**a**) Perspective view of TX coils; (**b**) Perspective and side view of RX coil. The figure shows 3D modeling with a lumped model using Ansys Maxwell Electronics.

Figure 6 shows the simulation setup from the bottom of the transmitter, consisting of ferrite, three stacked TX coils, and a solenoid-type RX coil wrapped around a ferrite sheet. The dimensions and the number of turns of each TX coil is the same, and the specific size is in Table 2. The air gap is 20 mm based on TX coil 3 (blue) closest to the RX coil. The air gap between TX coil1 and RX coil is 24.56 mm, and the air gap between TX coil2 and RX coil is 22.28 mm. This difference is 2.28 mm, which is the diameter of the coil. 

When TX coil 1 and RX coil are aligned, θ is set to 0 degrees, and WPT and WTT simulations are performed at 30 degrees intervals from the xy-plane to 180 degrees clockwise from the RX coil. As in the analysis, to check whether the torque is proportional to the square of the current, the current of the TX coil is changed at intervals of 2A up to from 6 to 10A based on the maximum value and the torque is checked for one cycle. In order to find out the effect of the material difference between the transceivers, the electrical parameters were compared by putting the gap materials into tissues composed of water, air, muscle and fat, and skin, respectively. Finally, simulation was conducted to investigate the change in power transmission efficiency and torque according to load resistance.

### 3.2. Simulation Result

**Figure 7:**
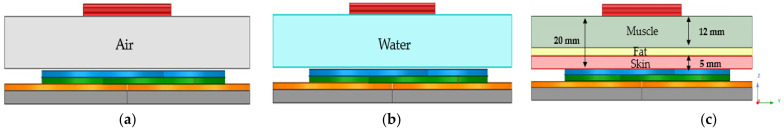
Simulation setup according to gap material. (**a**) Air; (**b**) Water; (**c**) Tissue made up of muscle, fat, and skin.

All three materials shown in Figure 7 have different conductivity, which can cause eddy currents to cause additional losses in the system [23]. When the conductivity is high, the coupling and inductance of the system are reduced, but in the case of human tissue, the conductivity is very low in the low frequency band, so there is little loss [24]. When TX coil 1 and RX coil are aligned, there is almost no difference as shown in Table 3 when comparing inductance and coupling. Therefore, when operating at a low frequency such as 125 kHz, the effect of the tissue is negligible.

**Figure 8:**
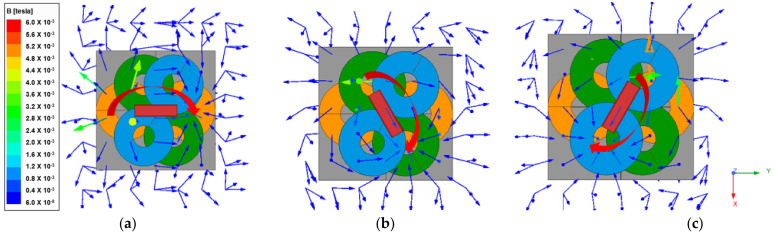
Simulation result of magnetic field vector direction and RX coil position according to TX coil on/off. (**a**) Magnetic field direction when TX coil 1 is on; (**b**) Magnetic field direction when TX coil 2 is on; (**c**) Magnetic field direction when TX coil 3 is on.

Since the proposed system uses a constant AC current compensation circuit, first, the current flowing through the TX coil is fixed as AC current with a peak value of 8 A. In Figure 8, the direction of the magnetic field vector rotates clockwise on the xy-plane when current flows sequentially in each TX coil.

**Figure 9:**
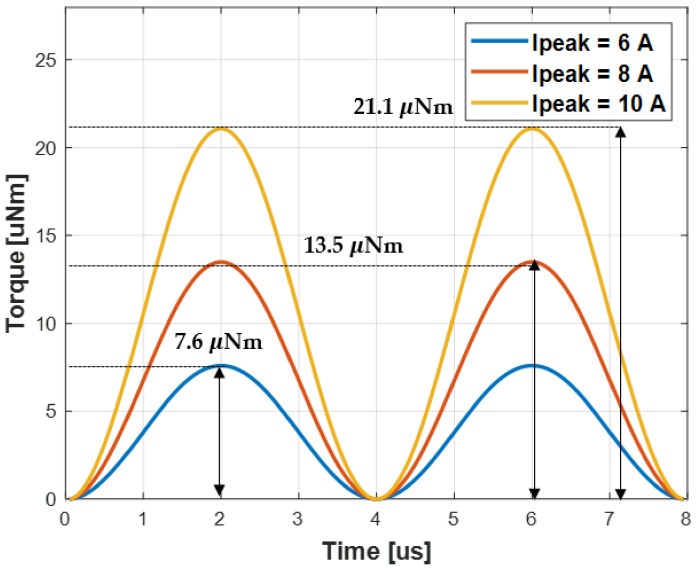
Torque per cycle generated in RX coil according to TX coil current size. The figure shows the torque result when the TX coil 1 differs by 60 degrees, and the torque increases in proportion to the square of the current.

In addition, in Equation (17), the torque is proportional to the square of the current, which is verified through simulation. Figure 9 shows that when the RX coil is aligned with the TX coil 1, the torque is simulated while changing the current flowing through the TX coil 2, and it can be confirmed that the torque proportional to the square of the current increase occurs. At this time, the current flowing through the TX coil is determined by the input DC voltage as shown in Equation (18).

As a result of WPT simulation, the DC to DC power transfer efficiency is shown in Table 4, where k is the coupling coefficient between the TX coil and the RX coil that generate torque. θ is the angle between TX coil 1 and RX coil. 

In Table 4, when θ is aligned with TX coil 1, TX coil 2 and TX coil 3, the θ is at 0 degrees, 60 degrees, and 120 degrees, respectively, and it means 60 degrees away from the coil that generates torque. So, it is when the physical distance is farthest. Therefore, the coupling coefficient and power transfer efficiency are the lowest at this time. On the other hand, the coupling coefficient and power transfer efficiency increase as the coil gets closer to the torque generating coil. In addition, since k is the same at 0 degrees and 180 degrees, the power transfer efficiency is also the same. The minimum transmitted power to the load is 1.7 W when θ is 60 degrees.

Table 5 shows the changes in system efficiency and torque when the load resistance is changed to 10 ohms and 50 ohms under the same conditions. As shown in Equation (22), the higher the load resistance, the lower the efficiency, and accordingly, the received power and the current flowing through the RX coil decrease. However, there is no significant difference in torque. This is because the torque is mainly determined by the current of the relatively large TX coil. As a result, the desired power can be received by adjusting the load resistance.

## 4. Experiments

The proposed method was verified through experiments, and the experiments were largely divided into WPT and WTT. First, the WPT experiment was conducted by placing the coil of the proposed structure by 20 mm of air gap between TX coil 3 and RX coil, and then placing the RX coil at intervals of 30 degrees.

For the WTT verification, the experiment was conducted by floating the RX coil as much as the air gap on the water. In order to implement an environment in which the z-axis of the receiving coil is fixed, an experiment was conducted on water, and torque generation was confirmed by turning on each TX coil sequentially.

### 4.1. Experiment Setup

**Figure 10:**
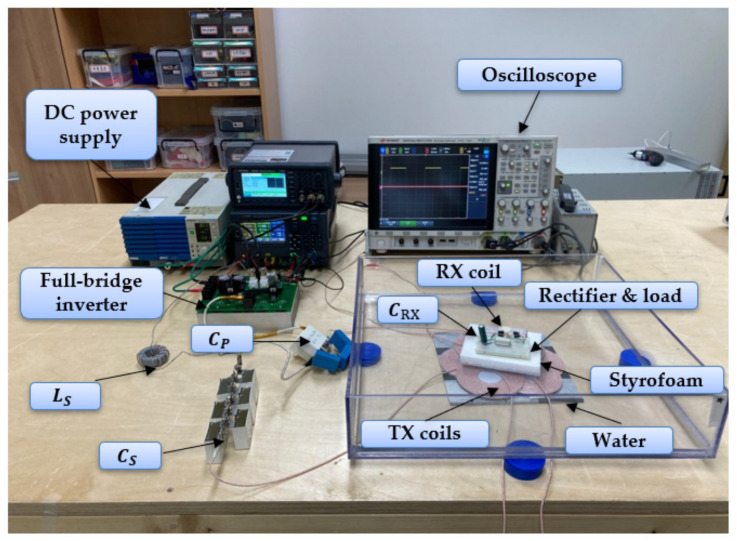
WTT and WPT experiment setup of the proposed models. The receiver, including the RX coil, capacitor, rectifier and load, is floated above the water.

Figure 10 shows the experimental setup to verify the proposed method. To verify the proposed WPT and WTT methods, the WPT system is first implemented. Transmitter including DC power supply, inverter and TX coils is out of the water tank and the receiver including RX coil, rectifier and load is in the tank. For the WTT experiment, for a stable measurement with the z-axis fixed, the experiment was carried out in in the air. However, to verify WTT, fill the water tank with water, float the receiver on the water, and check the torque generation.

Both the WPT and WTT experiments use the same system and use a DC power supply and inverter to transmit power to the TX coil and connect additional inductors and compensation capacitors for the circuit implementation suggested in Figure 4. Table 6 shows the inductors and capacitors used in the experiment. An oscilloscope is used to find the DC to DC efficiency by checking the current flowing through the TX and RX coils and measuring the voltage and current flowing through the load.

In addition, in order to analyze the WTT test result, after applying DC power, the rotation of the receiving coil was recorded as a video and used for analysis of the result.

Before the WPT and WTT experiment, to detect the position of the RX coil, it is checked whether the resistance reflected by the TX coil is seen as a measurement result depending on the position of the RX coil. After arranging the TX and RX coils in the proposed structure, calculating the load resistance reflected in the front end of the rectifier [25], connecting to the RX coil, rotating the RX coil at 30 degrees intervals, measuring the resistance of each TX coil using an impedance analyzer. Table 7 is the result of measuring the resistance of each TX coil according to the arrangement angle of the RX coil. $R_{\mathrm{Refl}1}$, $R_{R\mathrm{efl}2}$, and $R_{R\mathrm{efl}3}$ in Table 7 are the real components of the input impedance of TX coil 1, TX coil 2 and TX coil 3, respectively. As shown in Equation (24), the resistance of the TX coil increases as the mutual inductance increases in alignment with the TX coil. Therefore, in this experiment, even if the RX coil cannot be visually checked, it is conducted on the basis that it is possible to determine the TX coil to turn on by checking the position.

### 4.2. Experiment Result

**Figure 11:**
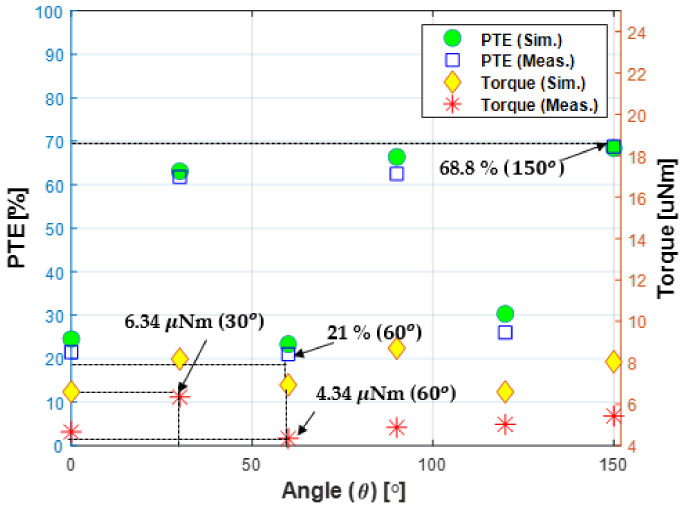
Simulation and experimental result of power transfer efficiency and torque of the proposed system. The power transfer efficiency varies from at least 21% to 68.8%, and the torque varies from 6.01 μNm to 8.78 μNm, which is relatively stable.

As a result of testing the proposed method, both DC to DC efficiency and torque for verifying WPT showed similar result to simulation result in Figure 11. First, the power transfer efficiency is at least 21% at 60 degrees, when TX coil3 is turned-on, and maximum 68.8% at 150 degrees, when TX coil1 is turned-on, similar to the simulation result. When the coupling coefficient with the TX coil is relatively low at 0 degrees, 60 degrees, or 120 degrees, the efficiency is low, and the efficiency increases as it approaches to the turned-on TX coil. In the experiment, between TX coil 2 and RX coil when θ is 0 degrees, between TX coil 2 and RX coil when θ is 30 degrees, between TX coil 3 and RX coil when θ is 60 degrees, between TX coil 3 and RX coil when θ is 90 degrees, between TX coil 1 and RX coil when θ is 120 degrees and between TX coil 1 and RX coil when θ is 150 degrees measure the efficiency and torque at each case.

**Figure 12:**
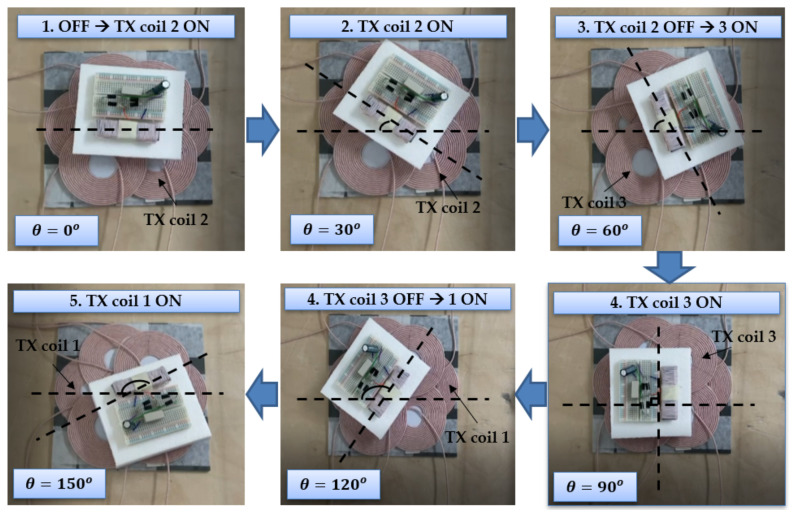
Torque generation process of RX coil according to turning on/off of TX coil. θ is the angle between TX coil 1 and RX coil. When the TX coil is turned on sequentially, the RX coil rotates clockwise to generate torque.

Torque is calculated using the equation in [12,26] by calculating the angular velocity and linear velocity using the video captured result. Specifically, the linear velocity in each case was obtained by playing the video and capturing the time it takes for the RX coil to rotate. The velocities at each position is given in Table 8. The torque experiment was carried out in the order shown in Figure 12. First, when all TX coils are OFF, TX coil 1, and RX coil are arranged in an aligned structure and the experiment is started. After that, when TX coil 2 is turned on to generate torque, the RX coil rotates clockwise until it aligns with TX coil 2 and is different from TX coil 1 by 60 degrees, then stops. After that, turn off TX coil 2 and turn on TX coil 3, and the RX coil will continue to rotate clockwise until 120 degrees aligned with TX coil 3. Finally, when TX coil 3 is turned off and TX coil 1 is turned on, the RX coil rotates until it aligns with TX coil 1 again. As a result of measuring the velocity at intervals of 30 degrees, the torque tends to increase as the TX coil and RX coil get closer, just like the efficiency.

As a result of the experiment, the torque was measured as 6.34 μNm when it rotates toward TX coil 2 and the angle differs from TX coil 1 by 30 degrees, and when it rotates toward TX coil 3 and the angle differs from TX coil 1 by 60 degrees, it is measured as 4.34 μNm. Therefore, the further the distance from the TX coil to generate the torque and the further away from the center of the coil where the magnetic field is the largest, the smaller the torque is generated. 

## 5. EMF Safety

Finally, an EMF safety analysis was performed to confirm that the proposed system complies with current standards. In general, the evaluation of the human body effect due to electromagnetic waves of the WPT system in the low frequency band of 10 MHz or less is interpreted by the magneto quasi-static method using a voxel-based human body model. The thermal effect caused by the electromagnetic field is evaluated by specific absorption rate (SAR), and is dominant in the frequency band above 10 MHz [27]. Therefore, according to the ICNIRP 2020 standard, SAR when the proposed system is inserted into the human body was resulted [28].

**Figure 13:**
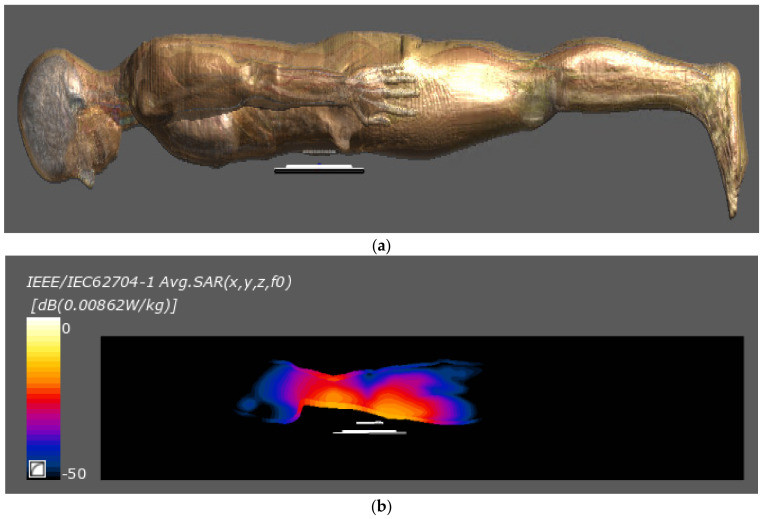
Simulation setup of the proposed models for SAR. (**a**) Simulation setup; (**b**) Simulation results of averaged local SAR.

To obtain the SAR, the induced electric field generated in the proposed WPT system was analyzed using Ella among the anatomical models of Information Technologies in Society (IT’IS). The simulation was carried out in the case of matching with TX coil3 among the positions of the RX coil, and the maximum received power was 26.5 W. As shown in Figure 13, the insertion position of the receiver is subcutaneously under the abdomen, and the transmitter is 20 mm away from the receiver.

In the case of local SAR in the operating frequency range of the proposed system as shown in Table 9, the simulation result shows 0.0086 W/kg, which is far below the standard of 2 W/kg [28]. At this time, local SAR is the average value of cubic with a mass of 10 g. Even the SAR of the maximum point is 0.0486, which is also lower than the limitation. Therefore, the EMF safety and temperature issues of the proposed WPT system are guaranteed as a result of the simulation.

## 6. Discussion

In this paper, we propose a method in which WPT and WTT are possible simultaneously. By using the magnetic resonance WPT system to transmit torque to the RX coil according to the magnetic field direction, power and torque can be transmitted at the same time. Therefore, this method can remove the battery of the drug pump in use and operate it, which It is very different from previous researches.

Through analysis and experiments, it was confirmed that current is generating magnetic field. Therefore, using the LCC-S topology that can keep the magnitude of the current constant, it was confirmed through experiments that a relatively constant torque was generated. 

The power transfer efficiency has a large range of change compared to the torque, but it is judged sufficient for the operation of the device, such as a micro flow meter, in that it can receive more power than necessary during the operating time. In addition, converter or regulator can be used to deliver power to the load as needed.

In the future, it is expected that the system will be manufactured with PCB and it will be possible to miniaturize it for children or animals if necessary.

In addition, high current flows through the TX coil located outside the body, so it is dangerous when it comes into contact with the human body. Therefore, in future, a separate housing is required using an insulator such as acrylic for patient safety.

## 7. Conclusions

In this paper, to solve the inconvenience of reoperation due to the use of batteries in implantable medical devices, we propose a method of simultaneously wireless power transfer and torque transfer. Specifically, conventional drug pump that drives a motor using the power of a battery to inject drugs into the body requires a heavy medical reoperation of reconnecting the device to the spinal cord when the battery life is ended. Therefore, in order to solve this problem, a system that can eliminate the battery itself and receive torque and power for communication or sensing is proposed at the same time. 

This method uses the principle of WPT using magnetic coupling between TX coil and RX coil. By turning on each TX coil in turn to change the direction of the magnetic field to the desired direction, the RX coil with magnetic material rotates and aligns, and torque is generated in the process. The drug pump requires stable torque generation, and for this, the current in the coil have to be relatively constant. Therefore, it uses the LCC-S topology to reliably supply torque and transmit power at the same time. 

In addition, in order to solve the problem of not being able to determine the position of the RX coil when it is inserted into the body, it is proposed that method of measuring the resistance of the TX coil reflected from the receiver. Through this, by measuring the parameters of the TX coil outside the body, the position of the RX coil can be easily found, and thus the TX coil to turn on can be determined.

Through the analysis, the proposed method was verified that WPT and WTT are possible at the same time through simulation and experiment. It was confirmed that a torque of 4.8 μNm or more required for the operation of the drug pump was generated, and it was also confirmed through an experiment that at least 1.5 W of power was delivered to the load when the received power efficiency was the lowest.

## Figures and Tables

**Table 1:** Electrical parameters of the circuit.

Parameters	Symbol	Parameters	Symbol
DC input voltage	$V_{DC}$	Resistance of TX coil	$R_{TX}$
Inverter output voltage	$V_{TX}$	Resistance of RX coil	$R_{RX}$
Load resistance voltage	$V_{L}$	Series inductance	$L_{S}$
Current flowing in $L_{s}$	$I_{S}$	Inductance of TX coil	$L_{TX}$
Current flowing in $C_{P}$	$I_{P}$	Inductance of RX coil	$L_{RX}$
Current flowing in $L_{TX}$	$I_{TX}$	Parallel capacitance	$C_{P}$
Current flowing in $L_{RX}$	$I_{RX}$	Series capacitance	$C_{S}$
Current flowing in $R_{L}$	$I_{L}$	Matching capacitance of RX coil	$C_{RX}$
Load resistance	$R_{L}$	Mutual inductance between TX coil and RX coil	$M_{TXRX}$

**Table 2:** Size specification of the proposed WTT and WPT system.

Size	Width [mm]	Length [mm]	Height [mm]
TX coils	-	-	2.28
RX coil	20	71.5	4.6
Ferrite (TX)	200	200	4
Ferrite (RX)	17.2	71.5	1.8
Air gap	-	-	20

**Table 3:** Electrical parameters depending on the gap material.

	Conductivity [s/m]	$L_{TX1}\;[\mathrm{uH}]$	$L_{RX}\;[\mathrm{uH}]$	$M_{TX1RX}\;[\mathrm{uH}]$
Air	0	52.597	27.959	4.805
Water	0.01	52.597	27.959	4.804
Tissue	Muscle 0.367 Fat 0.0434 Skin 0.00057	52.603	27.957	4.802

**Table 4:** Coupling coefficient and power transfer efficiency (PTE) of WTT and WPT system.

θ [deg]	0	30	60	90	120	150	180
k	0.042	0.094	0.042	0.1	0.05	0.11	0.042
PTE [%]	24.5	63.1	23.3	66.4	30.3	68.4	24.5

**Table 5:** Efficiency and torque according to load resistance.

$R_{L}$ [Ω]	$P_{in}\;[W]$	$P_{out}\;[W]$	PTE [%]	$I_{TX}\;[A]$	$I_{RX}\;[A]$	T [uNm]
10	9.3	2.52	26.9	5.6	0.56	6.425
20	8.6	2.12	24.5	5.6	0.33	6.424
50	7	0.75	10.7	5.6	0.15	6.431

**Table 6:** Value of parameters of the proposed system.

Coil	Type	Number of Turns	Inductance [uH]	$L_{S}\;[\mathrm{uH}]$	$C_{S}\;[\mathrm{nF}]$	$C_{P}\;[\mathrm{nF}]$	$C_{RX}\;[\mathrm{nF}]$	Resistance [mΩ]
TX coil 1 (Yellow)	Litz wire (2.28 mm)	28	52.9	11.3	37	143	-	170
TX coil 2 (Green)			47.9		41			140
TX coil 3 (Blue)			42.6		49			120
RX coil (Red)	Litz wire (1.3 mm)	47	30.3	-	-	-	54	160

**Table 7:** Measurement result of reflection resistance of TX coil according to RX coil angle.

θ [deg]	0	30	60	90	120	150	180
$R_{\mathrm{Refl}1}\;\left[m\Omega \right]$	470	422	280	170	222	409	460
$R_{R\mathrm{efl}2}\left[m\Omega \right]$	170	450	457	280	140	140	188
$R_{R\mathrm{efl}3}\left[m\Omega \right]$	250	116	120	300	453	350	220

**Table 8:** Angular velocity and linear velocity according to the receiving coil angle.

θ [deg]	0	30	60	90	120	150
$\omega_{t}$ **[rad/s]**	0.0748	0.0873	0.0722	0.0748	0.0776	0.0911
v **[cm/s]**	0.262	0.306	0.253	0.268	0.272	0.282

**Table 9:** Specifics absorption rate (SAR) of proposed model.

Frequency [kHz]	Local SAR [W/kg]		Max SAR [W/kg]
125	ICNIRP 2020	Proposed model	0.0468
	2	0.0086	

## Data Availability

The data presented in this study are available on request from the corresponding author.
